# COVID-19 Outcomes Among Persons Living With or Without Diagnosed HIV Infection in New York State

**DOI:** 10.1001/jamanetworkopen.2020.37069

**Published:** 2021-02-03

**Authors:** James M. Tesoriero, Carol-Ann E. Swain, Jennifer L. Pierce, Lucila Zamboni, Meng Wu, David R. Holtgrave, Charles J. Gonzalez, Tomoko Udo, Johanne E. Morne, Rachel Hart-Malloy, Deepa T. Rajulu, Shu-Yin John Leung, Eli S. Rosenberg

**Affiliations:** 1 New York State Department of Health, Albany; 2 Department of Health Policy Management and Behavior, University at Albany School of Public Health, State University of New York, Rensselaer; 3 Center for Collaborative HIV Research in Practice and Policy, University at Albany School of Public Health, State University of New York, Rensselaer; 4 Department of Epidemiology and Biostatistics, University at Albany School of Public Health, State University of New York, Rensselaer

## Abstract

**Importance:**

New York State has been an epicenter for both the US coronavirus disease 2019 (COVID-19) and HIV/AIDS epidemics. Persons living with diagnosed HIV may be more prone to COVID-19 infection and severe outcomes, yet few studies have assessed this possibility at a population level.

**Objective:**

To evaluate the association between HIV diagnosis and COVID-19 diagnosis, hospitalization, and in-hospital death in New York State.

**Design, Setting, and Participants:**

This cohort study, conducted in New York State, including New York City, between March 1 and June 15, 2020, matched data from HIV surveillance, COVID-19 laboratory-confirmed diagnoses, and hospitalization databases to provide a full population-level comparison of COVID-19 outcomes between persons living with diagnosed HIV and persons living without diagnosed HIV.

**Exposures:**

Diagnosis of HIV infection through December 31, 2019.

**Main Outcomes and Measures:**

The main outcomes were COVID-19 diagnosis, hospitalization, and in-hospital death. COVID-19 diagnoses, hospitalizations, and in-hospital death rates comparing persons living with diagnosed HIV with persons living without dianosed HIV were computed, with unadjusted rate ratios and indirect standardized rate ratios (sRR), adjusting for sex, age, and region. Adjusted rate ratios (aRRs) for outcomes specific to persons living with diagnosed HIV were assessed by age, sex, region, race/ethnicity, transmission risk, and CD4^+^ T-cell count–defined HIV disease stage, using Poisson regression models.

**Results:**

A total of 2988 persons living with diagnosed HIV (2109 men [70.6%]; 2409 living in New York City [80.6%]; mean [SD] age, 54.0 [13.3] years) received a diagnosis of COVID-19. Of these persons living with diagnosed HIV, 896 were hospitalized and 207 died in the hospital through June 15, 2020. After standardization, persons living with diagnosed HIV and persons living without diagnosed HIV had similar diagnosis rates (sRR, 0.94 [95% CI, 0.91-0.97]), but persons living with diagnosed HIV were hospitalized more than persons living without diagnosed HIV, per population (sRR, 1.38 [95% CI, 1.29-1.47]) and among those diagnosed (sRR, 1.47 [95% CI, 1.37-1.56]). Elevated mortality among persons living with diagnosed HIV was observed per population (sRR, 1.23 [95% CI, 1.07-1.40]) and among those diagnosed (sRR, 1.30 [95% CI, 1.13-1.48]) but not among those hospitalized (sRR, 0.96 [95% CI, 0.83-1.09]). Among persons living with diagnosed HIV, non-Hispanic Black individuals (aRR, 1.59 [95% CI, 1.40-1.81]) and Hispanic individuals (aRR, 2.08 [95% CI, 1.83-2.37]) were more likely to receive a diagnosis of COVID-19 than White individuals, but they were not more likely to be hospitalized once they received a diagnosis or to die once hospitalized. Hospitalization risk increased with disease progression to HIV stage 2 (aRR, 1.29 [95% CI, 1.11-1.49]) and stage 3 (aRR, 1.69 [95% CI, 1.38-2.07]) relative to stage 1.

**Conclusions and Relevance:**

In this cohort study, persons living with diagnosed HIV experienced poorer COVID-related outcomes relative to persons living without diagnosed HIV; Previous HIV diagnosis was associated with higher rates of severe disease requiring hospitalization, and hospitalization risk increased with progression of HIV disease stage.

## Key Points

**Question:**

Is there an association between prior diagnosis of HIV infection and coronavirus disease 2019 (COVID-19) diagnosis, hospitalization, and in-hospital death among residents of New York State?

**Findings:**

In a cohort study of linked statewide HIV diagnosis, COVID-19 laboratory diagnosis, and hospitalization databases, persons living with an HIV diagnosis were more likely to receive a diagnosis of, be hospitalized with, and die in-hospital with COVID-19 compared with those not living with an HIV diagnosis. After demographic adjustment, COVID-19 hospitalization remained significantly elevated for individuals with an HIV diagnosis and was associated with elevated mortality.

**Meaning:**

Persons living with an HIV diagnosis experienced poorer COVID-related outcomes (principally, higher rates of severe disease requiring hospitalization) relative to those without an HIV diagnosis.

## Teaser

This cohort study evaluates the association between HIV diagnosis and coronavirus disease 2019 diagnosis, hospitalization, and in-hospital death in New York State.

## Introduction

Coronavirus disease 2019 (COVID-19) has resulted in more than 1.6 million deaths worldwide as of December 15, 2020, with the United States reporting the most diagnosed cases (n = 16 520 408) and deaths (n = 300 494).^1^ In addition to having an older, more male demographic distribution, persons living with diagnosed HIV infection have a higher prevalence of many underlying medical conditions associated with more severe COVID-19 illness.^2,3,4^

The Centers for Disease Control and Prevention identifies older adults and those with certain underlying medical conditions as being at elevated risk for severe illness from COVID-19. Persons living with diagnosed HIV infection with a low CD4 cell count or not receiving HIV treatment are currently listed by the Centers for Disease Control and Prevention as possibly at risk for severe illness from COVID-19.^5^ Little has been firmly established regarding the extent to which persons living with diagnosed HIV are acquiring COVID-19, the severity of COVID-19 illness experienced by persons living with diagnosed HIV, or how these distributions compare with persons without diagnosed HIV. Emerging literature suggests similar or better COVID-19 clinical outcomes for persons living with diagnosed HIV compared with persons living without diagnosed HIV.^4,6,7,8,9,10,11,12^ Most of these studies have been small and limited to hospitalized populations of persons living with diagnosed HIV, limiting generalizability and a more complete understanding of the population-based risk of severe COVID-19 disease requiring hospitalization. A few recent and larger studies have found increased hospitalization rates and mortality outcomes among persons living with diagnosed HIV.^13,14,15,16^

New York State (NYS) has been an epicenter for both the US COVID-19 and HIV/AIDS epidemics, placing the state in a position to describe the intersection of these 2 epidemics. As of December 15, 2020, NYS has 784 204 diagnosed cases of COVID-19 and leads the US in COVID-19 deaths, at 35 427.^1^ At the end of 2018 in the US, NYS ranked second in the number of persons living with diagnosed HIV and first in the rate of HIV per 100 000 population.^17^ Similar to NYS’s COVID-19 epidemic, HIV infection is concentrated in New York City (NYC), among older adults, and among racial/ethnic minorities; in 2018, 78% of persons living with diagnosed HIV in NYS lived in NYC, 55% were older than 50 years, and 72% were non-Hispanic Black (40%) or Hispanic persons (32%).^18^ A recent match of surveillance databases within NYC compared the features and outcomes of persons living with diagnosed HIV with those of persons living with diagnosed HIV with COVID-19, but it did not estimate outcome rates or adjust for confounding factors associated with both infections.^16^

We conducted a population-level match of NYS’s HIV registry against its COVID-19 diagnosis and hospitalization databases, to provide a full population-level comparison of COVID-19 outcomes between the persons living with diagnosed HIV and persons living with diagnosed HIV within a US jurisdiction. Specifically, we compared the continuum of rates of COVID-19 diagnoses, hospitalizations, and in-hospital deaths for persons living with diagnosed HIV in NYS with those for persons living without diagnosed HIV and assessed factors associated with these outcomes among persons living with diagnosed HIV.^19^

## Methods

### Study Population and Data Sources

This is a retrospective cohort study of individuals with polymerase chain reaction–confirmed severe acute respiratory syndrome coronavirus 2 (SARS-CoV-2) infection diagnosed between March 1 and June 7, 2020, in NYS. Data were abstracted from the following: (1) the NYS HIV surveillance registry, which receives name-based reports for all HIV-related laboratory test results for individuals who reside in or receive HIV-related care in NYS^20,21^; (2) the NYS Electronic Clinical Laboratory Reporting System (ECLRS), an electronic system for secure and timely transmission of reportable clinical laboratory information; and (3) the State Health Information Network for NY (SHIN-NY), a public health information exchange network connecting NYS health care institutions. The NYS Department of Health institutional review board approved this study as exempt research not requiring informed consent. Data were deidentified after matching and prior to analysis. This study followed the Strengthening the Reporting of Observational Studies in Epidemiology (STROBE) reporting guideline.

### Outcomes

COVID-19 diagnoses, hospitalizations, and in-hospital deaths were evaluated among persons living with diagnosed HIV and persons living without diagnosed HIV. People with a diagnosis of COVID-19 were identified from an ECLRS file of all polymerase chain reaction–confirmed SARS-CoV-2 infections reported to the NYS Department of Health. The subset of individuals with HIV and diagnosed COVID-19 were identified by matching records from the ECLRS file of confirmed cases of SARS-CoV-2 infection with the NYS HIV surveillance registry. Data were matched using a deterministic matching algorithm implemented in SAS DataFlux, version 2.7 (SAS Institute Inc), which is used to link all routine and supplemental matches with the NYS HIV surveillance registry.^22^ This algorithm was earlier validated against the probabilistic matching software previously used by the registry. Because HIV surveillance data may require up to 1 year to ensure quality and completeness of information for individuals who are newly diagnosed, persons living with diagnosed HIV residing in NYS as of December 2019 were included in these analyses.

COVID-19 hospitalizations are routinely identified by ongoing matching of ECLRS data with the SHIN-NY database.^23^ The SHIN-NY datafile contained hospital encounter data from January 1 to June 15, 2020, with admission dates 14 days prior to 30 days after a positive COVID-19 test result. Individuals were considered hospitalized because of COVID-19 if they had a positive COVID-19 test result 3 days or less after discharge, an admission date 30 days or less after a positive COVID-19 test result, or a positive COVID-19 test result during the hospital encounter period. In-hospital death was defined by discharge status codes of 20 to 21, 40 to 41, or 42, as defined by the HL7 (Health Level Seven), version 2.5 discharge disposition value set,^24^ or a discharge description indicative of patient death.

### Study Variables

The demographic variables included in all analyses were age, sex, and region of residence (Long Island, Mid-Hudson, NYC, and rest of NYS). For analyses among persons living with diagnosed HIV, race/ethnicity, HIV transmission risk at diagnosis, receipt of HIV-related care, stage of HIV infection at last test, and viral load suppression at last test were available. Viral load, CD4, and genotype tests in the 3 years prior to the study period were included for analysis. Receipt of HIV-related care was defined as having a CD4, viral load, or genotype test result reported to the HIV surveillance registry in the 365 days prior to March 1; HIV disease stage at last CD4 test (for those aged ≥6 years: stage 1, ≥500 cells/mm^3^; stage 2, 200-499 cells/mm^3^; and stage 3, <200 cells/mm^3^) and viral suppression less than 200 copies/mL at last test were evaluated in the previous 3 years.^25,26,27^

### Statistical Analysis

COVID-19 diagnoses, hospitalizations, and in-hospital deaths were evaluated between persons living with diagnosed HIV and persons without diagnosed HIV.^28^ Proportion, or attack rates, expressed per 1000 persons, as well as attack rates per previous outcome (hospitalized per diagnosis and in-hospital death per hospitalization) and unadjusted rate ratios (RRs) with 95% CIs were calculated to evaluate associations between study variables and each outcome. Adjusted comparisons were made via indirect standardization, controlling for age, sex, and region.^29^

Among persons living with diagnosed HIV, the attack rates per 1000 persons and per previous outcome, with unadjusted RRs with 95% CIs, were assessed among levels of age, sex, region, race/ethnicity, transmission risk, care status, HIV stage, and viral suppression status. Adjusted RRs (aRRs) with 95% CIs were calculated in multivariable Poisson regression models, which included covariates significant at α = .05 in bivariate analyses. Viral suppression was not included in multivariable models because it is a potential mediator of the association between HIV stage and COVID-19 outcomes. All analyses were conducted using SAS, version 9.4 (SAS Institute Inc).^22^ For these models, missing values were imputed for HIV transmission risk (11.9% [12 869 of 108 062]), race/ethnicity (0.1% [113 of 108 062]), and age (0.002% [2 of 108 062]) using a fully conditional specification, implemented using SAS PROC MI.^30,31^ Multiple imputation was not implemented for laboratory variables with missing data. All *P* values were from 2-sided tests and results were deemed statistically significant at *P* < .05.

## Results

From March 1 to June 7, 2020, among 108 062 persons living with diagnosed HIV in NYS, 2988 (2109 men [70.6%]; 2409 living in New York City [80.6%]; mean [SD] age, 54.0 [13.3] years) received a diagnosis of COVID-19 at a rate of 27.7 per 1000, which was higher than among persons living without diagnosed HIV (rate, 19.4 per 1000; RR, 1.43 [95% CI, 1.38-1.48]) (Table 1). Similarly, elevated rates of COVID-19 were observed across age categories (except for persons aged 40-59 years), sex, and region of residence at HIV diagnosis. Standardization for these factors yielded an overall adjusted diagnosis rate ratio (sRR) of 0.94 (95% CI, 0.91-0.97), comparing persons living with diagnosed HIV with persons living without diagnosed HIV (Figure). Standardized RRs were significantly above 1.0 in regions outside of NYC but lower in NYC (eTable 1 in the Supplement).

**Table 1.** COVID-19 Diagnosis, Hospitalization, and In-Hospital Death per 1000 Individuals, Among PLWDH and Non-PLWDH—New York State, March 1 to June 7, 2020^a^

Characteristic	Population size, No.		Diagnosed					Hospitalized					In-hospital death				
	PLWDH^b^	Non-PLWDH	PLWDH		Non-PLWDH		Rate ratio (95% CI)	PLWDH		Non-PLWDH		Rate ratio (95% CI)	PLWDH		Non-PLWDH		Rate ratio (95% CI)
			No.	Rate per 1000	No.	Rate per 1000		No.	Rate per 1000	No.	Rate per 1000		No.	Rate per 1000	No.	Rate per 1000	
Age, y^c^																	
<40	27 154	9 902 345	492	18.12	121 871	12.31	1.47 (1.35-1.61)	62	2.28	7346	0.74	3.08 (2.40-3.95)	4	0.15	254	0.03	5.74 (2.14-15.42)
40 to <60	53 632	4 925 972	1400	26.10	133 095	27.02	0.97 (0.92-1.02)	356	6.64	15 925	3.20	2.05 (1.85-2.28)	67	1.25	1952	0.39	3.15 (2.47-4.02)
≥60	27 274	4 517 182	1096	40.18	119 291	26.41	1.52 (1.43-1.62)	478	17.53	38 096	8.38	2.08 (1.90-2.27)	136	4.99	12 316	2.71	1.83 (1.55-2.17)
Sex^d^																	
Female	30 331	9 975 384	879	28.98	182 440	18.29	1.59 (1.48-1.69)	265	8.74	27 366	2.74	3.19 (2.82-3.59)	66	2.18	5802	0.58	3.74 (2.94-4.77)
Male	77 731	9 370 115	2109	27.13	190 537	20.33	1.33 (1.28-1.39)	631	8.12	33 881	3.59	2.25 (2.08-2.43)	141	1.81	8699	0.92	1.95 (1.65-2.31)
Region of residence^e^																	
Long Island	5709	2 827 816	252	44.27	79 874	28.25	1.56 (1.38-1.77)	53	9.28	12 113	4.27	2.17 (1.66-2.84)	13	2.28	2666	0.94	2.42 (1.40-4.17)
Mid-Hudson	6142	2 317 635	228	37.24	61 771	26.65	1.39 (1.22-1.59)	46	7.49	6619	2.85	2.62 (1.96-3.51)	5	0.81	1089	0.47	1.73 (0.72-4.17)
New York City	84 284	8 252 524	2409	28.58	204 903	24.83	1.15 (1.11-1.20)	771	9.15	38 964	4.67	1.94 (1.80-2.08)	186	2.21	9995	1.20	1.82 (1.58-2.11)
Rest of New York State	11 916	5 947 524	88	7.37	23 395	3.93	1.88 (1.52-2.32)	25	2.10	3558	0.60	3.51 (2.37-5.20)	3	0.25	742	0.12	2.02 (0.65-6.27)
Total	108 062	19 345 499	2988	27.65	375 260	19.40	1.43 (1.38-1.48)	896	8.29	61 371	3.15	2.61 (2.45-2.79)	207	1.92	14 522	0.75	2.55 (2.22-2.93)

Abbreviations: COVID-19, coronavirus disease 2019; PLWDH, persons living with diagnosed HIV.

^a^ Persons with a diagnosis of COVID-19 through June 7, 2020, hospitalized through June 15, 2020.

^b^ Persons living with diagnosed HIV infection as of the end of December 2019, per data as of July 2020. Two persons had unknown age and 11 were living in New York State as of the end of 2019 but living out of state at COVID-19 diagnosis, classifying them as out of state for region of residence.

^c^ Age was at diagnosis for those with diagnosed COVID-19. For individuals with diagnosed HIV but not diagnosed COVID-19, age as of December 31, 2019, was used. Individuals younger than 18 years are included in the group who are younger than 40 years. Among non-PLWDH, 1003 diagnosed and 4 hospitalized had unknown age.

^d^ Sex at birth was used for PLWDH, sex for non-PLWDH may include current gender identity. Among non-PLWDH, 18 diagnosed and 1 hospitalized had “other” sex; 2265 diagnosed, 123 hospitalized, and 21 with in-hospital death had unknown sex.

^e^ Region of residence was defined as follows: For persons with and without HIV with a diagnosis of COVID-19, region was defined based on the county of residence at COVID-19 diagnosis. For persons with an HIV diagnosis, but not with a diagnosis of COVID-19, region was based on the last known county of residence recorded in the HIV surveillance registry as of year-end 2019. Among non-PLWDH, 3875 diagnosed, 36 hospitalized, and 7 with in-hospital death were out-of-state residents; 1442 diagnosed, 81 hospitalized, and 23 with in-hospital death had “unknown” region of residence.

**Figure.**
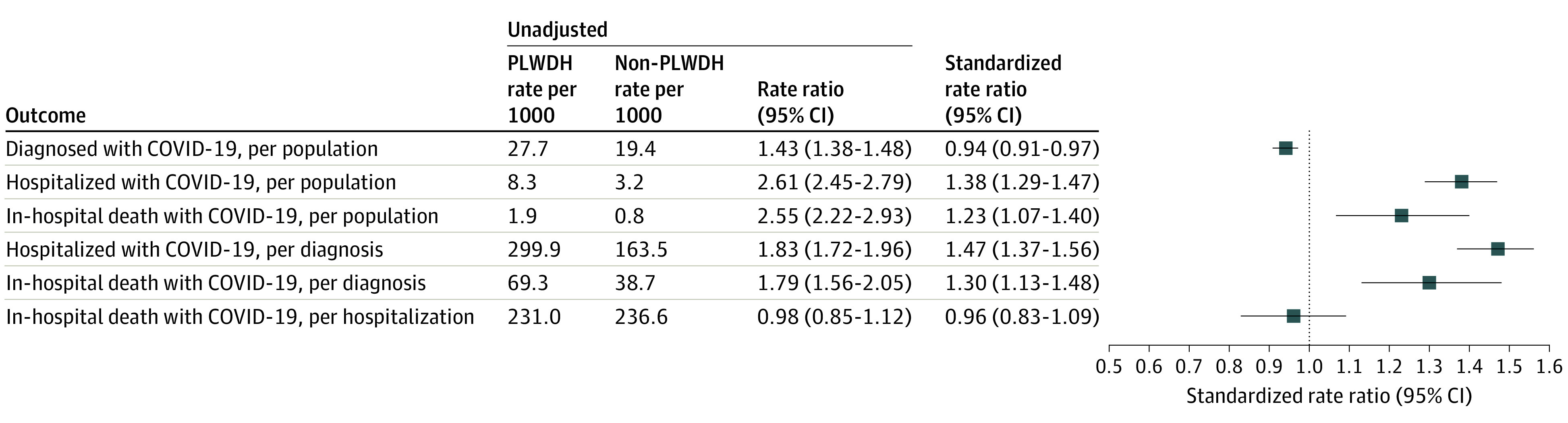
Summary of Rates and Rate Ratios for Coronavirus Disease 2019 (COVID-19) Diagnosis, Hospitalization, and In-Hospital Death, Comparing Persons Living With or Without Diagnosed HIV Infection, by Region—New York State, March 1 to June 7, 2020^a^ Standardized rate ratios are adjusted for sex, age, and region. PLWDH indicates persons living with diagnosed HIV. ^a^Persons with a diagnosis of COVID-19 through June 7, 2020, hospitalized through June 15, 2020. Standardized rate ratios adjusted for age, sex, and region.

Population-level rates of COVID-19 hospitalization were significantly elevated among persons living with diagnosed HIV (8.29 per 1000) vs persons living without diagnosed HIV (3.15 per 1000; RR, 2.61 [95% CI, 2.45-2.79]) and consistently so across age, sex, and geography (Table 1). In unadjusted analyses, relative hospitalization for persons living with diagnosed HIV vs persons living without diagnosed HIV was highest for persons younger than 40 years (RR, 3.08 [95% CI, 2.40-3.95]), women (RR, 3.19 [95% CI, 2.82-3.59]), and those living in the rest of NYS (RR, 3.51 [95% CI, 2.37-5.20]). After standardization, the disparity in hospitalization between persons living with diagnosed HIV and persons living without diagnosed HIV decreased but remained significantly elevated, per population (sRR, 1.38 [95% CI, 1.29-1.47]), and among those diagnosed with COVID-19 (sRR, 1.47 [95% CI, 1.38-1.56]).

Overall, 207 persons living with diagnosed HIV (rate, 1.92 per 1000) had a COVID-19 diagnosis and died in the hospital at a higher rate than persons living without diagnosed HIV (RR, 2.55 [95% CI, 2.22-2.93]) (Table 1). Unadjusted per-population relative mortality among persons living with diagnosed HIV vs persons living without diagnosed HIV was highest among persons younger than 40 years (RR, 5.74 [95% CI, 2.14-15.42]), women (RR, 3.74 [95% CI, 2.94-4.77]), and residents of Long Island (RR, 2.42 [95% CI, 1.40-4.17]). After adjustment, the standardized mortality ratio among persons living with diagnosed HIV vs persons living without diagnosed HIV was 1.23, per population (95% CI, 1.07-1.40), and was 1.79 (95% CI, 1.56-2.05) among those diagnosed.

The conditional rates per previous outcomes stage for persons living with diagnosed HIV vs persons living without diagnosed HIV are summarized in the Figure, alongside population-level rates, and in eTable 2 in the Supplement. Among those with a diagnosis of COVID-19, nearly one-third (299.87 per 1000) of persons living with diagnosed HIV were hospitalized, a higher rate than among persons living without diagnosed HIV (RR, 1.83 [95% CI, 1.72-1.96]). Among those hospitalized with COVID-19, no differences were seen in in-hospital death between persons living with diagnosed HIV and persons living without diagnosed HIV (RR, 0.98 [95% CI, 0.85-1.12]; sRR, 0.96 [95% CI, 0.83-1.09]). Despite the lack of significant difference in adjusted in-hospital mortality conditional on hospitalization, the higher levels of hospitalization for persons living with diagnosed HIV underpinned the significantly higher mortality rates per person and per diagnosis (case fatality rate, 69.28 per 1000 vs 38.70 per 1000; sRR, 1.30 [95% CI, 1.13-1.43]).

Among persons living with diagnosed HIV, in bivariate analyses, COVID-19 diagnosis was associated with all factors examined, except for sex (Table 2).^25^ In an adjusted model, persons living with diagnosed HIV of older age, not white non-Hispanic race/ethnicity, and living in the regions of metropolitan NYC were significantly more likely to receive a diagnosis of COVID-19. Among persons living with diagnosed HIV, non-Hispanic Black individuals (aRR, 1.59 [95% CI, 1.40-1.81]) and Hispanic individuals (aRR, 2.08 [95% CI, 1.83-2.37]) were more likely to receive a diagnosis of COVID-19 than White individuals, but they were not more likely to be hospitalized once they received a diagnosis or to die once hospitalized. No significant differences were observed between the main HIV transmission risk groups. After controlling for these factors, we found that having stage 3 HIV infection (vs stage 1: aRR, 1.22 [95% CI, 1.07-1.38]) was associated with increased rate of COVID-19 diagnosis.

**Table 2.** Factors Associated With Stages of COVID-19 Diagnosis, Hospitalization, and In-Hospital Death Among Persons Living With Diagnosed HIV Infection—New York State, March 1 to June 7, 2020^a^

Characteristic	Population size^b^	Diagnosed				Hospitalized				In-hospital death		
		No.	Rate per 1000 PLWDH	Rate ratio (95% CI)		No.	Rate per 1000 diagnoses	Rate ratio (95% CI)		No.	Rate per 1000 hospitalized	Unadjusted rate ratio (95% CI)
				Unadjusted	Adjusted^c^			Unadjusted	Adjusted^c^			
Age, y												
<40	27 154	492	18.12	1 [Reference]	1 [Reference]	62	133.50	1 [Reference]	1 [Reference]	5	80.65	1 [Reference]
40 to <60	53 632	1400	26.10	1.44 (1.30-1.60)	1.39 (1.24-1.54)	356	253.30	2.02 (1.54-2.64)	1.86 (1.40-2.46)	68	191.01	2.92 (1.06-7.99)
≥60	27 274	1096	40.18	2.22 (1.99-2.47)	2.09 (1.86-2.35)	478	441.80	3.46 (2.66-4.51)	3.09 (2.33-4.09)	134	280.33	4.41 (1.63-11.92)
Sex												
Female	30 331	879	28.98	1.07 (0.99-1.16)	NA	265	300.00	1.01 (0.87-1.16)	NA	66	249.06	1.12 (0.84-1.50)
Male	77 731	2109	27.13	1 [Reference]	NA	631	299.20	1 [Reference]	NA	141	223.45	1 [Reference]
Region of residence at diagnosis												
Long Island	5709	252	44.27	1.54 (1.36-1.76)	1.63 (1.42-1.86)	53	212.80	0.66 (0.49-0.87)	0.74 (0.56-0.99)	13	245.28	1.02 (0.58-1.78)
Mid-Hudson	6142	228	37.24	1.30 (1.13-1.49)	1.28 (1.11-1.47)	46	206.70	0.63 (0.47-0.85)	0.65 (0.47-0.89)	5	108.70	0.45 (0.19-1.09)
New York City	84 284	2409	28.58	1 [Reference]	1 [Reference]	771	317.90	1 [Reference]	1 [Reference]	186	241.25	1 [Reference]
Rest of New York State	11 916	88	7.37	0.26 (0.21-0.32)	0.27 (0.22-0.34)	25	270.30	0.89 (0.60-1.32)	0.99 (0.65-1.51)	3	120.00	0.50 (0.16-1.56)
Race/ethnicity												
Non-Hispanic												
White	20 563	337	16.39	1 [Reference]	1 [Reference]	86	255.20	1 [Reference]	1 [Reference]	20	232.56	1 [Reference]
Black	43 143	1146	26.56	1.62 (1.44-1.83)	1.59 (1.40-1.81)	364	317.60	1.25 (0.98-1.57)	1.15 (0.89-1.48)	85	233.52	1.00 (0.62-1.63)
Hispanic	35 914	1256	34.97	2.13 (1.89-2.41)	2.08 (1.83-2.37)	371	295.40	1.16 (0.92-1.46)	1.11 (0.87-1.43)	92	247.98	1.07 (0.66-1.72)
Not listed above^d^	8329	246	29.54	1.80 (1.53-2.12)	1.86 (1.57-2.20)	75	304.90	1.20 (0.88-1.63)	1.20 (0.87-1.66)	10	133.33	0.57 (0.27-1.23)
HIV transmission risk												
Heterosexual	30 578	913	29.86	1.32 (1.21-1.44)	1.01 (0.92-1.12)	257	281.50	1.19 (1.00-1.42)	0.98 (0.82-1.16)	62	241.25	1.12 (0.78-1.61)
IDU	11 432	439	38.40	1.70 (1.52-1.89)	1.12 (0.99-1.26)	190	432.80	1.83 (1.52-2.21)	1.13 (0.93-1.37)	55	289.47	1.34 (0.92-1.95)
MSM	46 741	1059	22.66	1 [Reference]	1 [Reference]	250	236.10	1 [Reference]	1 [Reference]	54	216.00	1 [Reference]
MSM and IDU	4118	130	31.57	1.39 (1.16-1.67)	1.16 (0.96-1.39)	48	369.20	1.56 (1.15-2.13)	1.13 (0.83-1.53)	7	145.83	0.68 (0.31-1.48)
Other	2324	27	11.62	0.51 (0.35-0.75)	0.57 (0.39-0.85)	4	148.10	0.63 (0.23-1.69)	0.89 (0.33-2.41)	NA	0.00	NA
Unknown	12 869	420	32.64	NA	NA	147	350.00	NA	NA	29	197.28	NA
Receipt of HIV care, previous 12 mo^e^												
Yes	93 704	2834	30.24	1 [Reference]	NA	844	297.80	1 [Reference]	NA	196	232.23	1 [Reference]
No	14 243	154	10.81	0.36 (0.30-0.42)	NA	52	337.70	1.13 (0.86-1.50)	NA	11	211.54	0.91 (0.50-1.67)
Stage of HIV Infection, at last test^f^												
Stage 1	63 712	1774	27.84	1 [Reference]	1 [Reference]	437	246.30	1 [Reference]	1 [Reference]	94	215.10	1 [Reference]
Stage 2	27 905	843	30.21	1.08 (0.99-1.18)	1.02 (0.94-1.11)	298	353.50	1.44 (1.24-1.66)	1.29 (1.11-1.49)	71	238.26	1.11 (0.81-1.51)
Stage 3	7498	270	36.01	1.29 (1.14-1.47)	1.22 (1.07-1.38)	126	466.70	1.89 (1.55-2.31)	1.69 (1.38-2.07)	34	269.84	1.26 (0.85-1.86)
Other	8947	101	11.29	NA	NA	35	346.50	NA	NA	8	228.57	NA
Viral suppression, at last test^f^												
Yes	87 480	2628	30.04	1 [Reference]	NA	756	287.70	1 [Reference]	NA	180	238.10	1 [Reference]
No	12 027	267	22.20	0.74 (0.65-0.84)	NA	105	393.30	1.37 (1.11-1.68)	NA	21	200.00	0.84 (0.54-1.32)
Other	8555	93	10.87	NA	NA	35	376.30	NA	NA	6	171.43	NA
Total	108 062	2988	27.65	NA	NA	896	299.87	NA	NA	207	231.03	NA

Abbreviations: COVID-19, coronavirus disease 2019; IDU, injection drug use; MSM, men who have sex with men; NA, not applicable.

^a^ Persons with a diagnosis of COVID-19 through June 7, 2020, hospitalized through June 15, 2020.

^b^ Persons living with HIV infection diagnosed as of the end of December 2019, per data as of July 2020. Two persons had unknown age, 11 were living in New York State at the time of HIV diagnosis but living out of state at COVID-19 diagnosis, classifying them as out of state for region of residence, 113 had unknown race/ethnicity, 115 had unknown receipt of care status (New York City cases not linkable to the statewide HIV registry). Individuals younger than 18 years are included in the group who are younger than 40 years.

^c^ Model adjusted for age, region of residence, race/ethnicity, HIV transmission risk (other includes pediatric, blood products), and stage at last test in the 3 years before March 1, 2020. Viral suppression is not included in multivariable models with stage of HIV infection, since viral suppression is a likely mediator of the association between HIV stage and COVID-19 outcomes.

^d^ Includes individuals who identify as Asian, Pacific Islander, Native American, or multiracial. There were insufficient data available to present analyses separately for each group.

^e^ Receipt of care status is based on any laboratory report received by the New York State HIV surveillance registry in the 12 months prior to March 1, 2020.

^f^ Stage and viral suppression are based on most recent test received by the New York State HIV surveillance registry in the 36 months prior to the study period. Stage of HIV infection was based on the Centers for Disease Control and Prevention case definition for adults and children, where stage 1 is CD4 of 500 cells/mm^3^ or more or 26% or more of total lymphocytes (aged ≥6 years) or CD4 of 1000 cells/mm^3^ or more or 30% or more of total lymphocytes (aged 1-5 years); stage 2 is CD4 of 200 to 499 cells/mm^3^ or 14% to 25% of total lymphocytes (aged ≥6 years) or CD4 of 500 to 999 cells/mm^3^ or 22% to 29% of total lymphocytes (age 1-5 years); and stage 3 is CD4 less than 200 cells/mm^3^ or less than 14% of total lymphocytes (aged ≥6 years) and CD4 less than 500 cells/mm^3^ or less than 22% of total lymphocytes (aged 1-5 years).^25^ Persons classified as “other” include those out of care for more than 36 months, who have moved out of state, and who have not moved but are receiving care out of state.

On further examination of the risk factors for hospitalization among persons living with diagnosed HIV and with diagnosed COVID-19, in adjusted analyses, older age and region, but not race and not ethnicity or transmission risk, were associated with hospitalization (Table 2).^25^ Relative to stage 1 infection, there was a gradient of increased hospitalization risk across stage 2 infection (aRR, 1.29 [95% CI, 1.11-1.49]) and stage 3 infection (aRR, 1.69 [95% CI, 1.38-2.07]). Among those hospitalized, only older age was associated with in-hospital death.

To probe the role of HIV stage in increasing hospitalization risk for persons living with diagnosed HIV vs persons living without diagnosed HIV, we conducted the per-person hospitalization standardized rate analysis by stage of HIV disease. Relative to persons living without diagnosed HIV, hospitalization risk was elevated for those with HIV stage 1 infection (sRR, 1.19 [95% CI, 1.08-1.30]), stage 2 infection (sRR, 1.60 [95% CI, 1.42-1.78]), and stage 3 infection (sRR, 2.66 [95% CI, 2.20-3.13]).

## Discussion

To our knowledge, our study represents the first population-level match of an entire US state’s HIV registry against its COVID-19 diagnosis and hospitalization databases, establishing state-level rates of COVID-19 outcomes among persons living with diagnosed HIV and comparisons with those observed in the overall population.

### COVID-19 Diagnosis

A total of 2.8% of persons living with diagnosed HIV in NYS (2988 of 108 062) had received a diagnosis of COVID-19 through June 7, 2020, nearly 40% higher than observed among persons living without diagnosed HIV. This disparity disappeared after standardization, consistent with a meta-analysis of 14 smaller studies (8 from the US) finding higher but nonsignificant rates of COVID-19 diagnoses among persons living with diagnosed HIV^32^ and with a recent NYC study.^16^ After adjustment, COVID-19 diagnosis rates among persons living with diagnosed HIV did not differ by sex at birth or transmission risk. Diagnosis rates were significantly higher among persons living with diagnosed HIV aged 40 years or older, a finding reported elsewhere.^33^ Consistent with a convenience sample of persons living with diagnosed HIV,^2^ our study found a higher than background rate of COVID-19 among persons living with diagnosed HIV of color; the adjusted diagnosis rates were 1.6 times higher for non-Hispanic Black individuals and 2.1 times higher for Hispanic individuals compared with non-Hispanic White individuals. This finding parallels increased levels of HIV diagnosis in NYS for persons of color^18^ and may reflect the association of differential rates of COVID-19–enhancing comorbidities among persons living with diagnosed HIV of color and/or social and behavioral determinants of health associated with COVID-19 transmission in minority communities.^34,35,36,37^

COVID-19 diagnosis rates among persons living with diagnosed HIV also varied by region. Consistent with overall population rates, we found significantly lower diagnosis rates among persons living with diagnosed HIV in upstate New York (rest of NYS). Diagnosis rates among persons living with diagnosed HIV were significantly higher in the NYC-adjacent regions of Long Island and Mid-Hudson than in NYC. This finding may reflect lower COVID-19 testing availability in NYC during the initial phase of the pandemic rather than a difference in background infection levels. Although there were no overall differences in COVID-19 diagnoses between persons living with diagnosed HIV and persons living without diagnosed HIV, persons living with diagnosed HIV exhibited higher rates of COVID-19 outside NYC and a lower rate within NYC. Because we were not able to standardize this comparison by race/ethnicity, this finding may reflect differences in the racial/ethnic distribution of persons living with diagnosed HIV relative to the overall population by region.

The association between the management of HIV infection and COVID-19 diagnosis was not straightforward because persons living with diagnosed HIV with viral suppression were significantly more likely to have received a diagnosis of COVID-19, as were persons living with diagnosed HIV with CD4 counts less than 200 cells/mm^3^.^3^ The increased diagnosis probability among persons with viral suppression may reflect a difference in test-seeking behavior or more interaction with the health care system among persons living with diagnosed HIV with viral suppression rather than a difference in underlying COVID-19 prevalence. Alternatively, healthier persons living with diagnosed HIV may be acquiring COVID-19 at higher rates, although this interpretation is inconsistent with 1 study.^3^

### COVID-19 Hospitalization and Mortality

We found that persons living with diagnosed HIV were significantly more likely than persons living without diagnosed HIV to be hospitalized with COVID-19, overall and among individuals with a diagnosis of COVID-19, suggesting higher rates of severe disease among persons living with diagnosed HIV requiring hospitalization. Hospitalization rates among persons living with diagnosed HIV were higher among those without viral suppression and those with lower CD4 counts, suggesting that more advanced disease may increase COVID-19 severity to the point that hospitalization is required. This interpretation is consistent with an NYC study showing elevated levels of hospitalization, intensive care unit admission, and death among persons living with diagnosed HIV, with lower CD4 count associated with poorer COVID-19 outcomes.^16^ Our finding that higher hospitalization rates among persons living with diagnosed HIV compared with persons living without diagnosed HIV persisted among the subset of persons living with diagnosed HIV who had high CD4 counts suggests that additional factors may be associated with elevated hospitalization rates among persons living with diagnosed HIV, including other comorbidities, systemic stress of chronic infection, and social determinants of COVID-19 severity. Hospitalization rates were also higher among those older than 40 years and those with documented history of injection drug use; the latter finding was also observed in a national, non–HIV-focused case-control study.^38^

We observed elevated population-level mortality for persons living with diagnosed HIV that was associated with higher hospitalization rates but not higher mortality among hospitalized persons living with diagnosed HIV. The unadjusted case fatality rate among persons living with diagnosed HIV was nearly twice that exhibited among persons living without diagnosed HIV, with a significant difference maintained but attenuated after statistical adjustment. The number of COVID-19 deaths among persons living with diagnosed HIV constitutes a sizable increase over normal levels. There were 490 deaths among persons living with diagnosed HIV from March 1 to June 15, 2019 (Mark Rosenthal, MSPH, NYS Department of Health, email, October 27, 2020). Against this backdrop, the 207 COVID-19–specific hospital deaths in our study represent a 42% addition to anticipated deaths during this same interval in 2020. Further analyses refining this estimate are needed.^39^ Higher mortality among persons living with diagnosed HIV was reported in a large population cohort study of health care attendees in South Africa,^13^ in cohorts of hospitalized patients in London^14^ and NYC,^15^ and in a study of persons living with diagnosed HIV receiving antiretroviral therapy in 60 Spanish hospitals.^33^ Contrary findings have been reported in an NYC study comparing 88 hospitalized persons living with diagnosed HIV with COVID-19 with a matched control group of persons living without diagnosed HIV.^4^

The only significant factor associated with in-hospital mortality among hospitalized persons living with diagnosed HIV was age, with those aged 40 years or older being 3 to 4 times more likely to experience in-hospital death. Given the well-established findings on elevated mortality by increasing age regardless of HIV status,^13,33,40^ this finding likely reflects an elevated risk of COVID-19 severity–enhancing comorbidities, including diabetes, hypertension, and chronic lung and cardiovascular disease, among older adults. Also, as reported in the literature, we found that persons living with diagnosed HIV hospitalized with and dying from COVID-19 were younger than persons living without diagnosed HIV.^12,41^ This finding may lend support to the notion that HIV infection can accelerate biological aging.^42,43^

Although non-Hispanic Black persons living with diagnosed HIV and Hispanic persons living with diagnosed HIV were more likely to receive a diagnosis of COVID-19 than non-Hispanic White persons living with diagnosed HIV, they were not more likely to be hospitalized once diagnosed or to die once hospitalized. This finding is partially consistent with COVID-19 studies finding racial/ethnic disparity present in hospitalization rates but not in mortality.^19,44,45,46^ Finally, despite RRs in the expected direction, CD4 count was not significantly associated with in-hospital death. This finding is incongruent with at least 2 studies finding that CD4 counts less than 200 cells/mm^3^ were significantly associated with decreased survival among hospitalized persons living with diagnosed HIV.^13,33^

### Limitations

This study has some limitations. The earliest outcome in this study is COVID-19 laboratory-confirmed diagnosis and not infection. A statewide seroprevalence study estimated that approximately 9% of COVID-19 cases through March 2020 had been diagnosed in NYS.^47^ Differences in diagnosis propensity among persons living with diagnosed HIV or between persons living with diagnosed HIV and persons living without diagnosed HIV could alter the interpretation of some findings. Our analyses were limited to the demographic and laboratory data available in NYS’s HIV surveillance registry and COVID-19 database, precluding a more in-depth understanding of the role played by comorbidities and underlying medical conditions, COVID-19 risk behaviors, and social determinants of health, which necessitates more comprehensive data sources, such as medical record reviews; however, the gradient in severity observed by CD4-defined HIV stage suggests a direct role for HIV disease. It is important to further investigate how the observed associations may be changed by information on comorbidities and underlying medical conditions, COVID-19 risk behaviors, and social determinants of health. Our denominator of persons living with diagnosed HIV included people who died between January 1 and June 15, 2020, and excluded persons who received a new diagnosis of HIV during this same time frame. Because these numbers have historically offset each other, the association of this limitation with outcomes is likely negligible.

## Conclusions

Although the mechanisms underpinning increased risk are not fully understood, the intersection of HIV and COVID-19 has multiple implications. Because HIV infection is a marker for, and may play a direct role in, more severe COVID-19 outcomes, persons living with diagnosed HIV (with any CD4 count) may warrant recategorization from “might be at increased risk” to “increased risk” in the Centers for Disease Control and Prevention’s underlying medical conditions list.^5^ This change may lead to higher prioritization of persons living with diagnosed HIV for receipt of the COVID-19 vaccine, per national and state allocation plans.^48,49^ Finally, a syndemic association between these infections may act multiplicatively on affected persons and communities, which are more likely to involve persons of color and urban areas.^50^ Our findings present an opportunity to address health equity with regard to HIV and COVID-19 through a combination of prevention and treatment approaches.^51^

## Notes

Supplement. **eTable 1.** Age- and Sex-Standardized Rate Ratios for COVID-19 Diagnosis, Hospitalization, and In-Hospital Death, Comparing Persons Living With and Without Diagnosed HIV Infection, by Region—New York State, March 1-June 7, 2020 **eTable 2.** COVID-19 Diagnosis, Hospitalization, and In-Hospital Death per 1000, Among Persons Living With and Without Diagnosed HIV Infection—New York State (NYS), March 1-June 7, 2020
