# Supplementary material for: COVID-19 Outcomes Among Persons Living With or Without Diagnosed HIV Infection in New York State
Source: JAMA Netw Open. 2021 Feb 3;4(2):e2037069. doi: 10.1001/jamanetworkopen.2020.37069 (PMC7859843; doi:10.1001/jamanetworkopen.2020.37069)
Supplement: Supplement. — eTable 1. Age- and Sex-Standardized Rate Ratios for COVID-19 Diagnosis, Hospitalization, and In-Hospital Death, Comparing Persons Living With and Without Diagnosed HIV Infection, by Region—New York State, March 1-June 7, 2020 eTable 2. COVID-19 Diagnosis, Hospitalization, and In-Hospital Death per 1000, Among Persons Living With and Without Diagnosed HIV Infection—New York State (NYS), March 1-June 7, 2020 [file jamanetwopen-e2037069-s001.pdf]

Supplementary Online Content

Tesoriero JM, Swain CAE, Pierce JL, et al. COVID-19 outcomes among persons living with or without diagnosed HIV infection in New York State. *JAMA Netw Open.* 2021;4(2):e2037069. doi:10.1001/jamanetworkopen.2020.37069

eTable 1. Age- and Sex-Standardized Rate Ratios for COVID-19 Diagnosis, Hospitalization, and In-Hospital Death, Comparing Persons Living With and Without Diagnosed HIV Infection, by Region—New York State, March 1-June 7, 2020
eTable 2. COVID-19 Diagnosis, Hospitalization, and In-Hospital Death per 1000, Among Persons Living With and Without Diagnosed HIV Infection—New York State (NYS), March 1-June 7, 2020

This supplementary material has been provided by the authors to give readers additional information about their work.

© 2021 Tesoriero JM et al. *JAMA Network Open.*

eTable 1. Age- and Sex-Standardized Rate Ratios for COVID-19 Diagnosis, Hospitalization, and In-Hospital Death, Comparing Persons Living With and Without Diagnosed HIV Infection, by Region—New York State, March 1-June 7, 2020 [a]

	**Diagnosed with COVID-19**	**Hospitalized with COVID-19**	**In-hospital Death with COVID-19**
	**Standardized Rate Ratio (95% CI)**	**Standardized Rate Ratio (95% CI)**	**Standardized Rate Ratio (95% CI)**
**Rates per 1,000 population**			
Long Island	1.33 (1.17-1.50)	1.64 (1.20-2.08)	1.80 (0.82-2.79)
Mid-Hudson	1.14 (1.00-1.30)	1.89 (1.34-2.43)	1.23 (0.15-2.31)
New York City	0.88 (0.85-0.92)	1.32 (1.23-1.41)	1.20 (1.03-1.37)
Rest of New York State (ROS)	1.81 (1.43-2.19)	2.91 (1.77-4.06)	1.85 (0.00-3.95)
**Rates per previous stage** [b]			
Long Island	1.34 (1.17-1.50)	1.27 (0.93-1.62)	1.09 (0.50-1.68)
Mid-Hudson	1.15 (1.00-1.30)	1.50 (1.07-1.94)	0.68 (0.08-1.28)
New York City	0.90 (0.86-0.93)	1.47 (1.37-1.58)	0.96 (0.83-1.10)
Rest of New York State (ROS)	1.81 (1.44-2.19)	1.80 (1.09-2.50)	0.81 (0.00-1.72)

a. Persons diagnosed with COVID-19 through June 7, hospitalized through June 15

b. Denominator for "Diagnosed with COVID-19" is population, for "Hospitalized with COVID-19" is persons diagnosed with COVID-19, for "In-hospital Death with COVID-19" is persons hospitalized with COVID-19.

© 2021 Tesoriero JM et al. *JAMA Network Open.*

eTable 2. COVID-19 Diagnosis, Hospitalization, and In-Hospital Death per 1000, Among Persons Living With and Without Diagnosed HIV Infection—New York State (NYS), March 1-June 7, 2020 [a]

	Population Size		Diagnosed with COVID-19					Hospitalized with COVID-19					In-hospital Death with COVID-19				
	PLWDH [b]	non-PLWDH	PLWDH		non-PLWDH			PLWDH		non-PLWDH			PLWDH		non-PLWDH		
			n	Rate/ 1,000	n	Rate/ 1,000	Rate Ratio (95% CI)	n	Rate/ 1,000 diag.	n	Rate/ 1,000 diag.	Rate Ratio (95% CI)	n	Rate/ 1,000 hosp.	n	Rate/ 1,000 hosp.	Rate Ratio (95% CI)
**Age in Years** [c]																	
<40	27,154	9,902,345	492	18.12	121,871	12.31	1.47 (1.35-1.61)	62	126.02	7,346	60.28	2.09 (1.63-2.68)	4	64.52	254	34.58	1.87 (0.69-5.01)
40-<60	53,632	4,925,972	1,400	26.10	133,095	27.02	0.97 (0.92-1.02)	356	254.29	15,925	119.65	2.13 (1.91-2.36)	67	188.20	1,952	122.57	1.54 (1.20-1.96)
60+	27,274	4,517,182	1,096	40.18	119,291	26.41	1.52 (1.43-1.62)	478	436.13	38,096	319.35	1.37 (1.25-1.50)	136	284.52	12,316	323.29	0.88 (0.74-1.04)
**Sex** [d]																	
Female	30,331	9,975,384	879	28.98	182,440	18.29	1.59 (1.48-1.69)	265	301.48	27,366	150.00	2.01 (1.78-2.27)	66	249.06	5,802	212.01	1.18 (0.92-1.50)
Male	77,731	9,370,115	2,109	27.13	190,537	20.33	1.33 (1.28-1.39)	631	299.19	33,881	177.82	1.68 (1.56-1.82)	141	223.45	8,699	256.75	0.87 (0.74-1.03)
**Region of Residence** [e]																	
Long Island	5,709	2,827,816	252	44.14	79,874	28.25	1.56 (1.38-1.77)	53	210.32	12,113	151.65	1.39 (1.06-1.82)	13	245.28	2,666	220.09	1.11 (0.65-1.92)
Mid-Hudson	6,142	2,317,635	228	37.12	61,771	26.65	1.39 (1.22-1.59)	46	201.75	6,619	107.15	1.88 (1.41-2.52)	5	108.70	1,089	164.53	0.67 (0.27-1.59)
New York City	84,284	8,252,524	2,409	28.58	204,903	24.83	1.15 (1.11-1.20)	771	320.05	38,964	190.16	1.68 (1.57-1.81)	186	241.25	9,995	256.52	0.94 (0.81-1.09)
Rest of NYS	11,916	5,947,524	88	7.39	23,395	3.93	1.88 (1.52-2.32)	25	284.09	3,558	152.08	1.87 (1.26-2.77)	3	120.00	742	208.54	0.58 (0.19-1.79)
**Total**	108,062	19,345,499	2,988	27.65	375,260	19.40	1.43 (1.38-1.48)	896	299.87	61,371	163.54	1.83 (1.72-1.96)	207	231.03	14,522	236.63	0.98 (0.85-1.12)

a. Persons diagnosed with COVID-19 through June 7, hospitalized through June 15.

b. Persons living with diagnosed HIV infection (PLWDH) as of the end of December 2019, per data as of July 2020. 2 persons had unknown age and 11 were living in NYS as of the end of 2019 but living out of state at COVID-19 diagnosis, classifying them as out of state for "region of residence".

c. Age was at diagnosis for those with diagnosed COVID-19. For individuals with diagnosed HIV but not diagnosed COVID-19, age as of 12/31/2019 was used. Individuals under age 18 are included in the "<40" group. Among non-PLWDH, 1,003 diagnosed and 4 hospitalized had unknown age.

d. Sex at birth was used for PLWDH, sex for non-PLWDH may include current gender identity. Among non-PLWDH, 18 diagnosed and 1 hospitalized had 'other' sex; 2,265 diagnosed, 123 hospitalized, and 21 with in-hospital death had unknown sex.

e. Region of residence was defined as follows: For persons with and without HIV diagnosed with COVID-19, region was defined based on the county of residence at COVID-19 diagnosis. For persons with an HIV diagnosis, but not diagnosed with COVID-19, region was based on the last known county of residence recorded in the HIV surveillance registry as of year-end 2019. Among non-PLWDH, 3,875 diagnosed, 36 hospitalized, and 7 with in-hospital death were out of state residents; 1,442 diagnosed, 81 hospitalized, and 23 with in-hospital death had 'unknown' region of residence.

© 2021 Tesoriero JM et al. *JAMA Network Open.*
